# Swimming Exercise in the Acute or Late Phase after Sciatic Nerve Crush Accelerates Nerve Regeneration

**DOI:** 10.1155/2011/783901

**Published:** 2011-08-21

**Authors:** Rosana Macher Teodori, Joice Betini, Larissa Salgado de Oliveira, Luciane Lobato Sobral, Sibele Yoko Mattozo Takeda, Maria Imaculada de Lima Montebelo

**Affiliations:** ^1^Master's Program in Physioterapy, Neuromuscular Plasticity Laboratory, FACIS, Methodist University of Piracicaba, 13400-911 Piracicaba, SP, Brazil; ^2^FACIS, Universidade Metodista de Piracicaba, 13400-911 Piracicaba, SP, Brazil

## Abstract

There is no consensus about the best time to start exercise after peripheral nerve injury. We evaluated the morphological and functional characteristics of the sciatic nerves of rats that began to swim immediately after crush nerve injury (CS1), those that began to swim 14 days after injury (CS14), injured rats not submitted to swimming (C), and uninjured rats submitted to swimming (S). After 30 days the number of axons in CS1 and CS14 was lower than in C (*P* < 0.01). The diameter of axons and nerve fibers was larger in CS1 (*P* < 0.01) and CS14 (*P* < 0.05) than in C, and myelin sheath thickness was lower in all crushed groups (*P* < 0.05). There was no functional difference between CS1 and CS14 (*P* > 0.05). Swimming exercise applied during the acute or late phase of nerve injury accelerated nerve regeneration and synaptic elimination after axonotmesis, suggesting that exercise may be initiated immediately after injury.

## 1. Introduction

Peripheral nerve injury promotes motor, autonomic, and sensory alterations in the region of the affected nerve, among which loss of function and progressive muscular atrophy stand out [1, 2]. Regeneration speed and subsequent functional recovery depend on the extension, nature, and degree of injury [3, 4]. In many cases morphological and functional recovery are not fully achieved [2], causing limitations in daily life and work activities [5], and may lead to early retirement due to functional disability. 

Several studies have investigated the effects of physical treatments on peripheral nerve regeneration and functional recovery, including phasic electrical stimulation [6–8], chronic low-frequency electrical stimulation [9, 10], ultrasound [11, 12], and physical exercise [8, 13–15]. Studies with rabbits after sciatic nerve crush showed that swimming exercise aids in both the removal of degenerated myelin and in its synthesis during nerve regeneration [16]. Physical exercise results in increased nerve impulse conduction speed and sensory-motor recovery [13] as well as in muscle property maintenance, assisting in trophism and minimizing muscle weakness after denervation [17]. However, there is no consensus on the ideal time to start exercise after denervation.

Considering that muscular reinnervation begins on the 14th day after injury, Herbinson et al. [18, 19] recommend that the stimulation of neuromuscular activity by exercise should begin approximately two weeks after nerve injury, leaving a rest period between the injury and the beginning of exercise. Gordon et al. [20] emphasized that, depending on the extent of the injury, exercise during the acute phase may reduce the number of motor units and axonal sprouting in extensively denervated muscles. On the other hand, it has been recommended that physical activity begin immediately after nerve injury or in the early stages of denervation to increase fatigue resistance and to restore the contractile properties and mechanical sensitivity of the muscle [21]. The resulting benefits have been reported to continue into the later phase of nerve regeneration [13] and functional recovery [14, 22]. Sobral et al. [15] compared the results of exercise on a treadmill in acute regeneration phases to those in late ones of the sciatic nerve of rats submitted to axonotmesis. They concluded that the exercise parameters neither negatively affected morphometric and functional recovery nor accelerated it.

Due to the lack of consensus about both the effects of exercise on the regeneration of peripheral nerve and the most appropriate time to begin intervention and also the need for greater understanding of the alterations involved in this type of injury, the objective of this study was to foster discussion about physiotherapeutic intervention by examining the influence of swimming exercise applied during acute (24 hours) and late (14 days) postlesion periods on the morphological and functional regeneration of the sciatic nerve in rats after axonotmesis.

## 2. Methods

This study was approved by the local Ethics Committee on Animal Experimentation. Twenty male Wistar rats, aged 6 to 7 weeks and weighing 220 ± 12 g, were obtained from the university's breeding colony and randomly divided into 4 groups (*n* = 5): swimming (S), Crush (C), crush + swimming 1st day (CS1), and crush + swimming 14th day (CS14). The S group was not injured but underwent the same exercise program as the CS1 group. The S group was not submitted to a sham-surgery procedure because it was assumed that the use of an analgesic would have controlled postoperative pain. The rats were maintained for 30 days in polyethylene cages with free access to water and commercial feed under controlled temperature and a light/dark cycle of 12/12 hours.

The animals submitted to swimming underwent an adaptation period in a 500 L, 60 cm high tank whose temperature was maintained at 32 ± 1°C. The adaptation phase lasted 20 minutes on the first day and was increased 10 minutes each day until reaching 60 minutes on the fifth day [22]. This adaptation period allowed the animals to become familiarized with swimming while avoiding both physical accommodation and stress [18, 23, 24], since stress can cause negative physiological, behavioral, and psychological changes in functional recovery after injury [13, 25].

After the adaptation period, the animals from C, CS1, and CS14 were anesthetized (ketamine chloride and xylazine chloride—0.045 mL/100 g and 0.03 mL/100 g of body weight, resp.). After trichotomy and asepsis, the left sciatic nerve was exposed by making a 15 mm incision in the gluteal region and crushed with an adapted haemostatic clamp in four 20 s clampings separated by 1 s intervals [7]. Muscle and cutaneous tissue were sutured with 6-0 Ethicon cotton thread. On the first two postoperative days, 4 *μ*L of the analgesic sodium dipyrone 500 mg/mL was administered every 12 hours.

After 24 hours (CS1) or 14 days (CS14) of nerve injury, the animals were submitted to group swimming, a more exciting and vigorous activity than individual swimming [26], with no additional load for 30 min each weekday for two weeks in the above-described tank filled with 40 cm of water, with a 24-hour interval established between each exercise session. At the end of each swimming session, the rats were dried with cloth towels and a hot hair dryer (Taiff RS-3) and replaced in their cages. The animals of S group underwent the same procedure, although they were not subjected to nerve injury.

To record the Sciatic Functional Index (SFI), animals from C, CS1, and CS14 groups were previously trained to walk on an 8.2 × 42 cm runway [27]. The runway was subsequently covered with white paper. The animals had their posterior paws stained with fingerprint ink and were made to walk [28]. Paw prints from both normal (uninjured) and experimental group animals were obtained in the preoperative period and at 7, 14, 21, and 28 postoperative (PO) days.

Using a digital paquimeter (*Mitutoyo*), the following distances for experimental (E) and normal (N) paws were obtained: the print length (PL) —between the end of the third toe and the calcaneous, total spreading (TS) —between the first and fifth toes, and Intermediary Toes (IT) —between the second and fourth toes [27, 29].

The obtained values were applied to the formula proposed by Bain et al. [29], where the results express functional loss as a percentage, with 0 (zero) representing normal function or absence of dysfunction and −100 (minus one hundred) representing total function loss.

After 30 days of injury, all animals were anesthetized according to the above-mentioned methodology, and the left sciatic nerve was exposed and fixed in situ at 4°C for 10 minutes with the modified Karnovsky [30] fixative containing 1% paraformaldehyde (Sigma, St Louis, Mo, USA) and 2% glutaraldehyde in sodium cacodylate buffer at 0.1 M, pH 7.3. The nerve was removed and a 5 mm segment distal to the injury was maintained in fixative solution (Karnovsky) for 24 hours and postfixed in 1% osmium tetroxide in sodium cacodylate buffer 0.1 M, pH 7.3 for two hours, immersed in 5% uranyl for 24 hours for en bloc staining, dehydrated in increasing solutions (30% to 100%) of acetone, and immersed in EPON solution. After nerve removal, the animals were euthanized by cervical dislocation.

Transversal slices (1 *μ*m) of the nerve were stained with 1% Toluidine blue in 1% aqueous borax solution and analyzed with an optical microscope (Olympus BX 41-BF) that was coupled to an image analysis system with Image Pro-Plus v6.2 software (Media Cybernetics). After obtaining the number of axons and the diameter of the nerve fibers and axons, the myelin sheath thickness and the G ratio were calculated.

The Shapiro-Wilk normality test was used. For morphometry, ANOVA-F (one-way) followed by the Tukey HSD test was applied. Since the SFI data were not normally distributed, the Friedman test was used for intragroup comparison and the Kruskal-Wallis test was used for intergroup comparison. The significance level was set at 5%.

## 3. Results

### 3.1. Quantitative Analysis

The number of axons was greater in C and CS1 than in S (*P* < 0.01); CS14 and S were similar. As shown in Table 1, the values in S, CS1, and CS14 were significantly lower than in C (*P* < 0.01).

### 3.2. Morphometric Analysis

As shown in Table 1, the mean axon diameter in C, CS1 and CS14 was lower than S (*P* < 0.01). Values in S, CS1, and CS14 were higher than in C (*P* < 0.01). The average fiber diameter in groups C, CS1, and CS14 was smaller than in S (*P* < 0.01), whereas in S, CS1, and CS14, the diameter was larger than in C (*P* < 0.05). The myelin sheath thickness in C, CS1, and CS14 was less than in S (*P* < 0.05). Values in S were higher than in C (*P* < 0.01). The G ratio values were significantly higher in CS1, and CS14 than in C and S (*P* < 0.01). However, all values were within the normal range.

### 3.3. Histological Analysis

**Figure 1:**
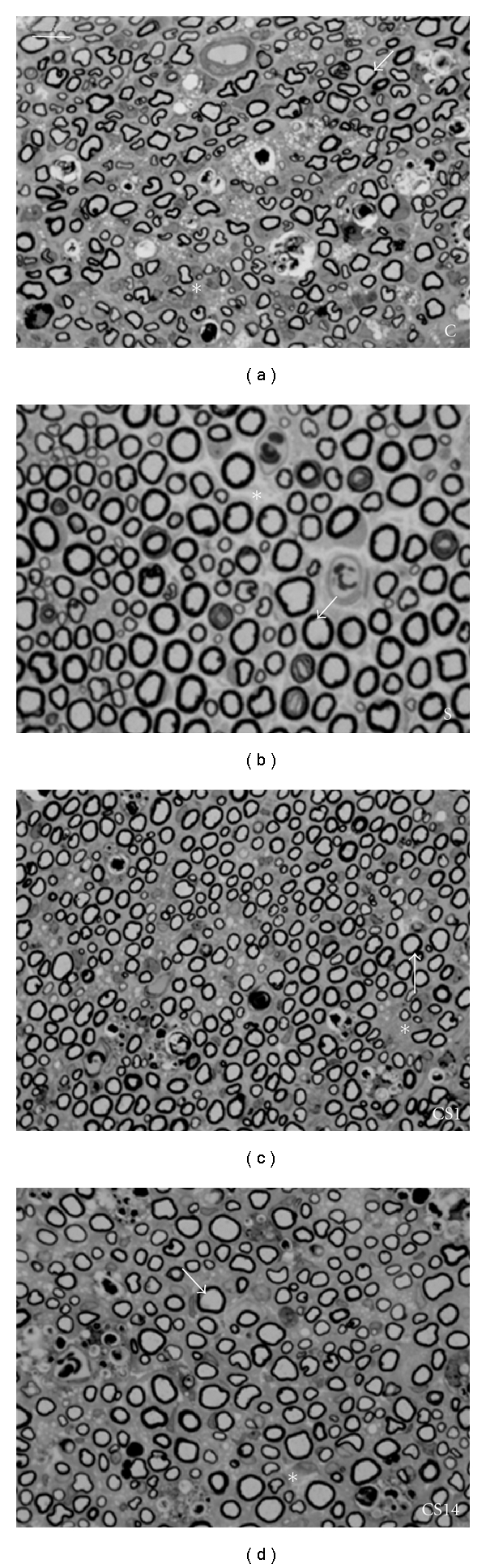
Cross section of sciatic nerve on groups Crush (C), Swimming (S), Crush + Swimming 1st day (CS1), and Crush + Swimming 14th day (CS14). Observe the axons (arrow) and the perineural tissue (*). Bar = 20 *μ*m.

In the S group, the caliber of the myelinated axons was consistent with that of normal sciatic nerve described in the literature and included myelin sheaths of normal thickness. In C, CS1, and CS14, the axons were smaller and had thinner myelin sheaths as well as more evident perineural tissue (Figure 1).

### 3.4. Functional Gait Analysis

All groups presented the same initial functional pattern. SFI values indicated normal function during the preoperative period. On the 7th and 14th PO days, these values decreased, indicating significant functional loss. Between the 21st and 28th PO days, the observed values suggested functional recovery. There was no significant difference between groups in different evaluation periods (*P* > 0.05). For intragroup comparison, there was a significant difference among the analysis periods. In C and CS14, the 7th and 14th PO days differed only from the preoperative period (*P* < 0.05), while in the CS1 group there was a difference between the 7th PO day and the preoperative period as well as between the 21st and 28th PO days and the 7th PO day (*P* < 0.05) (Table 2).

## 4. Discussion

The results of the present study show that swimming exercise applied during both the acute and late phases after axonotmesis in rats accelerated nerve fiber regeneration and synaptic elimination.

### 4.1. Number of Axons

All denervated groups presented two to three times more axons than controls. This can be explained by the fact that after nerve injury, each axon sprouts several branches [31] in the direction of the target organ. This number is only reduced when the target is reinnervated, a process known as synaptic elimination [31, 32].

Synaptic elimination begins after 26 days and is completed around the 60th day after sciatic nerve crush in rats that received no intervention [33]. The reduced number of axons in the animals submitted to exercise suggests that swimming may have accelerated synaptic elimination, which is an activity-dependent process [34] that favors monoinnervation with consequent recovery of physiological muscle activity, which was reaffirmed in this study by the presence of functional recovery. In this injury model, in which the nerve was analyzed after 30 days, a reduced number of axonal sprouts was observed far from the end plate (5 mm distal to nerve injury). According to Sanes and Lichtman [35], this may be due to the fact that regenerating axons reoccupy differentiated postsynaptic sites and that the synapses can mature more quickly than during normal nervous system development. Nevertheless, it should be observed that the maturation process was still ongoing after 30 days of injury, since the number of axons did not reach control values.

### 4.2. Morphometry

Regenerated axons with a reduced diameter are commonly found after peripheral nerve injury and are associated with a deprivation of terminal connections during regeneration, increased collagenation, endoneural retraction, and the late effects of injury on body cells [36]. Such a reduction was also observed in this study, since the recovery rates of CS1 and CS14 were 76.48% and 78.19%, respectively, of control values. However, denervated animals subjected to swimming presented axonal diameters 36 to 40% larger than the C group, regardless of when they began to exercise, which demonstrates that the proposed swimming protocol accelerated nerve regeneration.

Oliveira et al. [8], using the same intervention procedure, also observed an increase in the axonal diameter of rats subjected to swimming after axonotmesis. Nevertheless, they concluded that swimming did not accelerate the maturation of regenerated axons because they observed no difference in nerve fiber diameter compared to denervated rats not submitted to swimming. The differences between these results may be due to the difference in animal survival time, which was 22 days in their study.

Swimming exercise, whether in the acute or late phase of injury, also positively influenced the maturation of regenerated nerve fibers. After axonotmesis, axon diameters can reach control values after six months [37]. This, however, does not occur in nerve fibers, which reach only 75% of control values after injury [2]. In the present study, the nerve fiber diameter of groups CS1 and CS14 reached 68.07% and 70.22% of control values, respectively, while in the C group, it reached 56.8%. Considering that such results were obtained one month after injury, the benefits of swimming on nerve regeneration are evident.

The reduction of myelin sheath thickness observed in all denervated groups coincides with the results of Ansselin et al. [38], Verdú et al. [2], Oliveira et al. [8], and Sobral et al. [15]. Fraher and Dockery [36] indicated that the axon exerts control over myelin sheath thickness and that there is proportionality between the myelin sheath thickness and axon diameter. Sulaiman and Gordon [39] argue that the size of axons is regulated by the level of neurofilament expression in peripheral axons and that this expression is downregulated after axotomy and recovery when nerve-muscle contact is established. Although the axon and nerve fiber diameters were significantly higher in groups submitted to swimming exercise (CS1 and CS14) than in the injured-only group (C), the values of myelin sheath thickness did not differ from the C group. This may be due to the chronology of the nerve regeneration process, since, according to Burnett and Zager [40], the remyelination of regenerated axons begins 2 weeks after the onset of axonal regeneration. Thus, remyelination is a subsequent process that would have still not been completed, considering the survival time of animals in this study. Although the G ratio reached normal levels in all groups, the effects of swimming exercise on the maturation of regenerated axons can be considered positive.

The G ratio is a parameter related to nerve impulse conduction speed. It is a value obtained by the division of the axon's diameter by the nerve fiber diameter [38]. According to Torch et al. [41], G ratio values between 0.6 and 0.79 indicate normal nerve conduction speed. G ratio values below 0.6 indicate dense myelinization, while those above 0.79 indicate poor myelinization, both of which lead to conduction speed alteration. The results of this study suggest that swimming did not affect the conduction speed of nerve impulses, which remained within the normal levels after nerve regeneration.

Herbinson et al. [18] submitted rats with bilateral sciatic nerve injury to a protocol of swimming with a load (18 g). One group swam for 3 weeks, beginning 3 weeks after injury, and another group swam for 2 weeks, beginning 4 weeks after injury. They evaluated the reinnervation of the gastrocnemius muscle and concluded that exercise is more effective when started after four weeks of injury, stating that, when applied before this period, it inhibits reinnervation. Our results differ from theirs in that there were no morphometric or functional differences between the group that began exercise in the acute phase (24 hours after injury) and the one that began in the late phase (14 days after injury). Several factors may have contributed to this divergence, such as the time when exercise was initiated, bilateral denervation, and the use of a load during swimming, as well as the different types of analyses conducted in the studies.

### 4.3. Functional Recovery

The SFI values obtained in the preoperative period were in the normal range described by Dash et al. [42], which is between 0 and −20. SFI values near −100 on the 7th PO day show a complete loss of function due to denervation. Beginning on the 14th PO day, these values become less negative, which coincides with the onset of muscle reinnervation—when 25% of the muscle fibers are polyinnervated [33, 43]. On the 21st PO day, SFI values closer to zero were evident, showing that functional recovery reflects the polyinnervation peak which, according to Gorio et al. [33], occurs between the 21st and 25th day. On the 28th PO day, the values reflected a functional condition consistent with that of normal nerves due to continued regeneration and synaptic elimination until the occurrence of monoinnervation.

Despite similar trends among the groups in each period, when analyzing SFI values in each group from the preoperative period until the 28th PO day, it was observed that when activity began in the acute phase of injury, functional recovery was faster, something that was not observed in the other groups. Sarikcioglu and Oguz [16] suggest, moreover, that swimming exercise accelerates both the removal of degenerated myelin and its synthesis during the regeneration process, which provides improvement in the conduction speed of nerve impulses and subsequent functional recovery. However, in this study there was no significant difference between groups. It is interesting to note that, even when the morphological recovery of the nerve had not yet been fully achieved, functional recovery was observed. Burnett and Zager [40] emphasized that the return of function does not necessarily require a complete recovery of nerve architecture, a fact demonstrated in this study.

Sobral et al. [15] observed that exercise initiated in both the acute and late phases of nerve regeneration after axonotmesis neither benefitted nor harmed morphometric and functional recovery, arguing that exercise should begin while in the acute phase of regeneration since it can slow muscular atrophy. Byun et al. [14] subjected rats to daily exercise on a treadmill for 30 minutes and observed an acceleration of functional recovery after sciatic nerve crush. Oliveira et al. [8] studied the effects of 30 minutes of daily swimming during the acute phase of crush injury in rats and observed that this type of exercise did not damage nerve regeneration or functional recovery.

### 4.4. Neural Plasticity

It is possible that swimming directed the plasticity of the nervous system by cross-activation, seeing that denervation was unilateral and the animals were subjected to exercise that involved the bilateral action of members. In humans without nerve injury, sensory stimuli are processed primarily in the contralateral hemisphere, but there is also, to some extent, an ipsilateral activation [44–46]. In rats, the whisker region of the somatosensory cortex integrates information from both the contralateral and ipsilateral whisker pads [47, 48].

Considering the physiological aspect, it is likely that the tactile, thermal, and proprioceptive stimuli during swimming positively influenced nerve regeneration from cross-activation. Munn et al. [49] performed a meta-analysis to examine the effect of contralateral strength training from controlled studies in humans. They pointed out that unilateral training promotes increases in contralateral and ipsilateral limb strength of 7.8% and 35%, respectively, and that although the mechanisms involved in this process are not clearly defined, contralateral improvement seems to be related to neural mechanisms that involve excitation of relevant regions of the cerebral cortex during voluntary muscle contraction.

Kristeva et al. [50], in a study that assessed neuromagnetic fields during unilateral and bilateral voluntary human movements, also showed that unilateral voluntary movements promote activity in the contralateral motor cortex. Hortobágyi [51] reported that cross-activation is specific for homologous muscles in humans, since, after chronic strengthening with maximum voluntary contraction, the maximum voluntary contraction force of trained muscles increased from 30 to 40%, while the untrained contralateral homologous muscles showed an increase of 20% in maximum voluntary contraction. Kristeva et al. [50] mentioned that even submaximal contractions on one side of the body can produce contralateral homologous muscle activation.

Peripheral nerve injury is followed by profound long-term changes in cortical maps [43] as well as in several subcortical structures [52]. With respect to nerve crush injury, the reorganization of cortical maps is rapid [53], especially in rats, with a restoration of preexisting maps immediately after the regenerated axons have reinnervated their original targets [54]. Although an assessment of the cerebral cortex was not conducted in this study, something that should be the target of future investigations, it is possible that peripheral nerve regeneration was facilitated by the effect of swimming on central nervous system changes. Given an adequate stimulus, the central nervous system responds with changes such as increased synaptic connections and increased neuronal spine turnover [55–57], which may vary due to environmental influences as well as to the unmasking of silent synapses [46]. Such plastic alterations could increase the activation of lower motor neurons and, consequently, promote the synthesis of substances related to the regeneration of injured nerve fibers. Furthermore, the activation of denervated muscle promotes an increase in the density and blood flow of muscle capillaries [58], which maintain the metabolic conditions of muscle fibers and thus prevent atrophy and promote nerve regeneration [6, 59, 60]. Marqueste et al. [21] also pointed out that, in rats, exercise that begins during the acute injury phase increases resistance to fatigue and leads to recovery of the contractile and mechanic-sensitivity properties of muscle, which, according to Byun et al. [14] and Seo et al. [22], promotes functional recovery.

## 5. Conclusion

We conclude that swimming exercise applied both during the acute and late phases of crush injury accelerated rat sciatic nerve regeneration and synaptic elimination. These results should inspire new studies that reopen discussion of physiotherapeutic practice in related human treatment.

## Figures and Tables

**Table 1:** Mean values ± SE of the axon's number, axon's diameter (Ø axon), nerve fiber's diameter (Ø fiber), myelin thickness, and G ratio on swimming (S), crush (C), crush + swimming 1st day (CS1), and crush + swimming 14th day (CS14) groups.

Groups	Axon's number (un)	Ø Axon (*μ*m)	Ø fiber (*μ*m)	Myelin thickness (*μ*m)	G ratio
S	9709 ± 1453*	6.42 ± 0.54*	10.21 ± 0.87*	1.89 ± 0.24*	0.62 ± 0.02
C	21345 ± 2372^†^	3.60 ± 0.23^†^	5.80 ± 0.30^†^	1.09 ± 0.05^†^	0.62 ± 0.1
CS1	13954 ± 2035^∗†^	4.91 ± 0.20^∗†^	6.95 ± 0.29^∗†^	1.02 ± 0.06^†^	0.72 ± 0.04^∗†^
CS14	12730 ± 2467*	5.02 ± 0.29^∗†^	7.17 ± 0.48^∗†^	1.07 ± 0.13^†^	0.70 ± 0.02^∗†^

*differ from C group; ^†^differ from S group.

**Table 2:** Mean values ± SE of the SFI in the crush (C), crush + swimming 1st day (CS1) and crush + swimming 14th day (CS14) groups, in the different periods of analysis.

	C	CS1	CS14
Preoperative	− 16.18 ± 14.24	− 7.29 ± 20.37	− 9.78 ± 12.40
7th day	− 77.62 ± 25.17*	− 91.25 ± 30.20*	− 77.32 ± 25.46*
14th day	− 78.00 ± 13.66*	− 65.98 ± 37.22	− 72.13 ± 15.66*
21th day	− 20.20 ± 15.57	− 17.14 ± 11.72^†^	− 29.01 ± 12.12
28th day	− 14.66 ± 8.70	− 14.80 ± 12.91^†^	− 24.26 ± 17.31

*differ from preoperative; ^†^differ from 7th PO day (*P* < 0.05).
